# A Therapeutic Educational Program in Oral Health for Persons with Schizophrenia: A Qualitative Feasibility Study

**DOI:** 10.1155/2018/6403063

**Published:** 2018-09-24

**Authors:** Audrey Peteuil, Corinne Rat, Sahar Moussa-Badran, Maud Carpentier, Jean-François Pelletier, Frederic Denis

**Affiliations:** ^1^Instance Régionale d'Éducation et de Promotion de la Santé, 21000 Dijon, France; ^2^Clinical Research Unit, La Chartreuse Psychiatric Centre, 21033 Dijon, France; ^3^UFR Odontology and Public Health Department, 1 Avenue du Maréchal Juin, F-51095 Reims, France; ^4^Direction de la Recherche Clinique, University Hospital of Dijon, 21079 Dijon, France; ^5^Department of Psychiatry, Montreal University, Yale Program for Recovery and Community Health, Montreal, Canada; ^6^EA 75-05 Education, Ethique, Santé, Université François-Rabelais Tours, Faculté de Médecine, 37032 Tours, France

## Abstract

**Objective:**

The aim of this study was to test the feasibility of a therapeutic educational program in oral health (TEPOH) for persons with schizophrenia (PWS).

**Design:**

In a qualitative study, we explored the representation of oral health before and after a TEPOH. *Clinical Setting*: PWS are at greater risk of decayed and missing teeth and periodontal diseases. In a previous publication, we described the different steps in building a TEPOH by taking into account the experiences of PWS concerning oral health quality of life. This TEPOH aimed at promoting a global health approach. *Participants*: Voluntary PWS and their caregivers were recruited during face-to-face interviews at “Les Boisseaux” (a psychiatric outpatient centre) in Auxerre (France) and were included in the study between November and December 2016. *Intervention*: We explored the experiences of participants and their perceptions of oral health before and after the TEPOH with focus group meetings.

**Results:**

Four females and three males participated in the study, and the mean age was 29.4 ± 5. Before the TEPOH, the PWS produced 28 ideas about oral health perception and 37 after the TEPOH. After the TEPOH, elements relating to the determinants of oral health (smoking and poor diet) emerged.

**Conclusions:**

These results show an evolution in oral health representation, and after some adjustments to the TEPOH, the second step will be to test this program in a large sample to generate a high level of evidence of the impact of TEPOH in the long term.

## 1. Background

Analysis of the literature shows a gap in health between persons with severe mental illnesses, such as schizophrenia, and the general population. The life expectancy of people with severe mental illnesses is 15 to 20 years shorter than that of the general population, and they are more prone to excess morbidity [1]. In oral health, descriptive studies indicated that this population is also at a greater risk of developing tooth decay, missing teeth, and periodontal diseases [2]. Oral health as part of the general health of persons with schizophrenia (PWS) involves complex interactions between their mental illness, the social and medical support systems in which these people live, and the health care they receive [3]. PWS frequently do not recognize their health need along with the adverse effects of the different psychiatric treatments, like hyposalivation induced by antipsychotics [4] or hypersalivation with clozapine [5]. Other side effects do occur. First-generation antipsychotics can induce neurological effects (e.g., dystonia and dyskinesia), especially shaking, which prevents effective tooth brushing and impairs chewing and swallowing [6]. Second-generation antipsychotics tend to induce metabolic side effects, such as obesity or diabetes, rather than neurological effects [7]. Periodontal disease is associated with metabolic side effects [8], and it is now generally accepted that poor oral health is associated with chronic medical conditions such as myocardial infarction and stroke.

Evidence suggested that mental health risk factors may be associated with oral symptoms [9]. PWS are recognized as a priority group in need of support to improve oral health. However, Khokhar et al. noted that there are currently no studies supporting the need for routine clinical practices in this field [10].

A report by the Kings Fund (2016) in Australia “Bringing together physical and mental health: A new frontier for integrated care” argues that there needs to be a stronger focus on the integration of physical and mental health and that this aspect of integration should lead to the development of new models of care for all nurses and other health care professionals to address existing health inequalities [11].

In a previous publication, we described a study protocol to assess the effectiveness of a therapeutic educational programme in oral health (TEPOH) for PWS in France, with a cluster randomized controlled trial. We also explained the different steps necessary for building a TEPOH while accounting for the experiences of PWS in oral health quality of life and their active participation in the building process of the TEPOH. This TEPOH was aimed at promoting a global health approach and developing appropriate strategies to encourage multidisciplinary treatment of dental disorders, prospective support for PWS, and the development of training in oral health or mental health caregivers [12]. The TEPOH consisted of three workshops: an introductory session and a debriefing session each lasting 90 min and 2 weeks apart. The different themes of the workshops were mobilization of motivational approaches by improving self-esteem and well-being, called “Yes we can”. The second was demystifying dental surgery and was called “Even more afraid”. The third theme was improvement of oral health by a transverse approach to quality of life (cessation of smoking, controlling diabetes, management of good diet, etc.) and was called “Take care of myself” [12].

However, before assessing the effectiveness of this programme in a large sample, it was necessary to test the programme in real conditions with a cluster of PWS.

Therefore, the aim of this study was to test in a qualitative study the feasibility of the TEPOH through the evaluation of oral health in a sample of PWS.

## 2. Methods and Materials

### 2.1. Patients

Voluntary PWS (7) and their caregivers were recruited following face-to-face interviews at “Les Boisseaux” (a psychiatric outpatient centre) in Auxerre (France) and were included in the study between November and December 2016. The inclusion criteria were patients over 18 years of age with a diagnosis of schizophrenia, according to the Diagnostic and Statistical Manual of Mental Disorder-Fifth edition: DSM-5 [13]. Informed consent was obtained for participation in this study. The exclusion criteria were diagnosis other than schizophrenia, persons not stabilized from a psychiatric viewpoint, and patients with a decompensated organic disease and mental retardation. This decision was made by the psychiatrist of the patient. PWS who could not understand or had a poor understanding of French were excluded from the study. We confirm that participation was voluntary, that the participants could not be identified from the material presented, and that no plausible harm to participating individuals could result from the study. After providing participants with a complete description of the study, a written informed consent was obtained from each participant (or from their legal guardians for persons under guardianship).

The trial received ethical approval from the Committee for the Protection of Persons Number II of Eastern France (Approval reference: 2015-A00407-42).

### 2.2. Method

#### 2.2.1. Design

We used a focus group (FG) meeting to explore the evolution of the markers of oral health before and after the TEPOH.

First, we explained the TEPOH to the PWS and their caregivers. Second, we explored the experiences of the participants and the meaning of oral health for approximately 90 min using the following questions:

- What do you think about your oral health?
- Why you don't take care of your oral health?
- Are you afraid of the dentist?
- Do you think there is a link between oral health and dental health?

These open questions served to guide the interview and were selected from the qualitative study we conducted to build the TEPOH content with an expert group made up of health professionals, PWS, and PWS caregivers [12].

**Figure 1:**
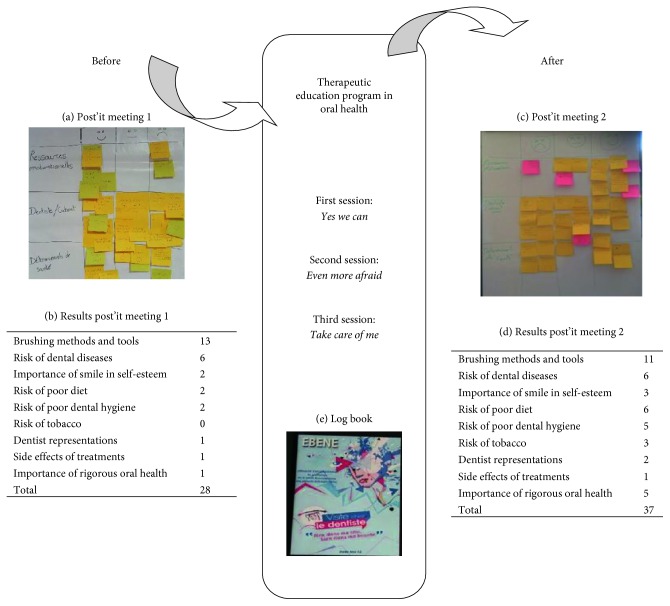
Evolution of the representations in oral health in a sample of PWS.

The post-it meeting technique was used to collect information and was classified into three categories on a paper board: positives, negatives, or neutral experiences about oral health (Figure 1).

According to researchers, positive assertions were assertions made by the PWS indicating ways to improve oral health. Negative assertions were assertions made by the PWS indicating a lack of proper oral hygiene. Neutral assertions involved other topics that emerged during the FG meeting. Group interactions encouraged respondents to provide insights that would not have surfaced during individual interviews. Participants were free to talk to other group members. The FG meeting covered personal data and insights that would have been less accessible without interactions in a group setting. The FG meeting was conducted by a specialist in therapeutic education. The caregiver did not participate in the FG meeting but was solely an observer. He assisted in creating a favourable environment during the study and participated in the debriefing process with the researchers.

Third, the same specialist in therapeutic education conducted the TEPOH sessions for two weeks. Full details of this specific TEPOH for PWS are published elsewhere [12].

Fourth, at the end of the TEPOH, the same methodology as that used in the first step was used to access the evolution of markers of oral health (Figure 1).

The audio recordings of all the FGs were analysed by a working group of researchers composed of a specialist in therapeutic education, a dentist, and a nurse specializing in mental health.

During this feasibility study, we tested the tools of the TEPOH, especially the log book for helping patients in their daily life to take care of their own oral health and the movie used to demystify dental consultations.

#### 2.2.2. Sample Size

In total, 7 PWS participated in the two FG meetings. Generally, FGs are composed of groups of 4 to 12 people. In this case, the sample size of the FG meetings was sufficient [14]. Guest et al. [15] suggested that a sample size of two to three FGs will likely capture at least 80% of the themes. Thus, 7 individuals were sufficient to assess the feasibility of the TEPOH and to offer complimentary data for some adjustments if necessary [16].

## 3. Results

Four females and three males participated in this study, and the mean age was 29.4 ± 5. Before the TEPOH, the PWS produced 28 ideas regarding oral health perception (Figure 1) and 37 after the TEPOH (Figure 1).

One caregiver from “Les Boisseaux” (a psychiatric outpatient centre) in Auxerre (France) was present during the course of the study. This ensured proper organization of the sessions (paper board, room meeting, etc.), and the caregiver also contributed to the creation of a friendly atmosphere, thus allowing the good progress of the FG meetings. The caregiver suggested the organization of two sessions in the future in order to encourage more PWS to use the log book.

Before the TEPOH, the most frequently cited positive elements were related to the methods and tools used for tooth brushing along with the risks of dental diseases (pain, infections, etc.). We have not identified any negative assertions. All participants were motivated to improve their oral health. At this step, the determinants of oral health were not spontaneously addressed.

After the TEPOH, the elements related to the methodology of tooth brushing were less cited (13 to 11) than at the inaugural session. Conversely, the determinants of oral health (tobacco and poor diet) emerged.

These results emphasize an evolution in oral health representation, which translates, as indicated by the content and the number of words produced, into an enlargement of the general knowledge concerning the subject.

Other topics emerged (neutral) such as the cost of dental treatment and the different payment methods, which was an emergent problem for PWS.

The log book was of little use in noting the patients' efforts to improve their oral health (Figure 1). Generally, the patients declared “I forgot to make it”. On the contrary, the movie was a good resource to help patients demystify the process of the dental consultation.

## 4. Discussion

This TEPOH aimed at supporting caregivers to help patients in improving their oral health and promoting a global approach to health, not only with regard to dental care-centred approach.

We found that PWS understand that oral health can be improved by reducing tobacco consumption, with a good diet, or a good dental hygiene and that oral health can contribute to the improvement of self-esteem and oral health-related quality of life.

In this study, we noticed the motivation of the PWS to improve this health problem. The caregiver of the “Les Boisseaux” psychiatric outpatient centre was implicated in this study and helped to create a good environment to promote the programme. A key component of this TEPOH will be the long-term participation and involvement of the different health teams in introducing TEPOH in their practices to support the current practices in oral health for PWS.

This pilot study confirmed that PWS have significant knowledge of oral health, as observed during the building stage of the TEPOH [12].

It is important to take into account the cost of dental care and to introduce in the TEPOH an explanation for the different possible dental services and financial resources for PWS. In France, medical and dental care costs are partly covered in cases of conservative and surgical dental care (70%) and prosthetics and orthodontics treatment (30 to 50%) by national health insurance and complementary health insurance or by PUMa (Protection Universelle Maladie) for people with low income levels (below 8723€ per year in 2017). PUMa is free [17]. An additional module will be introduced in the TEPOH.

Although the log book has been of little use in noting the efforts to improve oral health, the chapter of the log book with guidelines for accessing a dental office gave them the confidence to engage themselves and consult a dentist more frequently. The movie helped them to further demystify this process. In a previous study conducted by our group, we confirmed the importance of an integrative guide to improve access to primary care for the management of chronic diseases and health promotion among patients with severe mental illnesses [18].

## 5. Limitation

Regardless of the procedure used to obtain seven patients in this qualitative study, the cohorts were composed of volunteers who differed from a representative population of PWS. Indeed, their cognitive level and oral health status may probably be higher than those observed in a representative sample of PWS. Furthermore, taking into account the small sample size of the PWS and the implementation of only two FGs, we can assume that our results are probably not exhaustive.

## 6. Conclusions

The TEPOH showed the capacity to improve patient knowledge and patient questioning about oral health. After some adjustments, the second step will be to test this programme in a larger sample and over a longer period of time.

## Data Availability

The data analyzed during the current study are available from the corresponding author on request.

## Abbreviations

TEPOH: Therapeutic educational program in oral health
PWS: Persons with schizophrenia
FG: Focus group
PUMa: Protection Universelle Maladie.
